# Comparative computational study of sulfur-donor additives for stabilization of FAPbI_3_ perovskites

**DOI:** 10.55730/1300-0527.3779

**Published:** 2025-12-04

**Authors:** Ilnar NURGALIEV, Akbarxon HAMZAYEV, Murad MARASULOV, Zavkiddin JULLIEV, Akhmad OBLAKULOV, Nigmat ASHUROV

**Affiliations:** Institute of Polymer Chemistry and Physics, Academy of Sciences of the Republic of Uzbekistan, Tashkent, Uzbekistan

**Keywords:** FAPbI_3_ perovskites, additives, phase stabilization, surface passivation, density functional theory, molecular dynamics simulations

## Abstract

A multiscale computational investigation integrating density functional theory (DFT) and molecular dynamics (MD) simulations was conducted to elucidate the mechanisms through which sulfur-containing donor molecules stabilize the photoactive α-phase of formamidinium lead iodide (FAPbI_3_) perovskites. The binding energetics, charge-transfer behavior, and hydrogen-bonding interactions of thiourea (TU), thiosemicarbazide (TSC), thiocyanate (SCN^−^), and diethyldithiocarbamate (DTC) were systematically analyzed. DFT results revealed pronounced Pb–S coordination and multidentate hydrogenbonding, with binding energies following the trend SCN–>TSC>DTC>TU. Thermodynamic analysis demonstrated that these additives lower the Gibbs free energy difference, thereby stabilizing the black α-phase, with TSC, TU, and to a lesser extent DTC exhibitingthe most pronounced effects. Projected density of statesanalysis confirmed that TU and DTC effectively suppressed trap states near the band edges without introducing midgap defects. MD simulations demonstrated preferential adsorption of all S-donors on FAPbI_3_ (001) surfaces, forming three to four hydrogen bonds per frame and achieving adsorption energies up to −52 kJ·mol^−1^. These findings reveal a direct correlation between coordination strength, electronic coupling, and thermodynamic stabilization, establishing TU, TSC, and DTC as promising additives for improving the phase stability and electronic performance of FAPbI_3_-based perovskite solar cells.

## 1 Introduction

Formamidinium lead iodide (FAPbI_3_) in its photoactive black α-phase remains one of the most promising light-absorbing materials for high-efficiency perovskite solar cells (PSCs), owing to its near-ideal bandgap (≈1.48 eV), excellent thermal stability, and long carrier lifetimes. However, the practical utilization of α-FAPbI_3_ is hindered by its intrinsic phase instability; under ambient conditions and moderate temperatures, it readily transforms into the nonphotoactive yellow δ-phase, thereby severely degrading device performance and operational stability [1–6].

To overcome this challenge, various strategies have been explored, including compositional alloying, interface engineering, and additive-assisted crystallization. Among these, sulfur-containing molecular additives have emerged as a particularly effective class of stabilizing agents. Compounds such as thiourea (TU), thiosemicarbazide (TSC), thiocyanate (SCN^−^), dimethylthiourea (DMTU), methylthiourea (MeTU), and dimethyl sulfoxide (DMSO) have demonstrated the ability to coordinate with lead halide precursors, regulate nucleation kinetics, and enhance film morphology. Their functionality arises primarily from the lone-pair electrons on sulfur, which engage in Pb–S coordination, and from hydrogen-bonding interactions between amino or imino groups and halide ions or organic cations.

Among these S-donor molecules, TU remains the most extensively studied and effective additive. It not only suppresses the α–δ phase transition in FAPbI_3_ but also enhances crystallinity, mitigates trap-assisted recombination, and improves environmental stability. However, comparative analyses indicate that other S-donors—such as TSC and SCN^−^—can exhibit comparable or even stronger coordination tendencies while imparting distinct effects on surface passivation and film uniformity. For instance, TSC provides additional N-donor sites that can reinforce hydrogen-bonding networks, whereas SCNforms compact Pb–S–C–N linkages contributing to denser grain packing. Despite these promising findings, a comprehensive understanding of how the electronic structure, geometry, and binding energetics differ among these S-containing ligands remains incomplete.

Previous studies have demonstrated that TU can form Lewis acid–base adducts with PbI_2_ and hydrogen-bonded complexes with FA^+^ and MA^+^ cations, thereby modulating precursor equilibria and crystallization pathways [7–11]. Nevertheless, the mechanistic origin of its selectivity toward FAPbI_3_ over MAPbI_3_, as well as the quantitative relationship between molecular binding and macroscopic phase stability, has not been fully elucidated.

In this work, we employ a multiscale computational framework integrating density functional theory (DFT) and classical molecular dynamics (MD) to elucidate the interactions of TU and other S-donor additives—thiosemicarbazide (TSC), thiocyanate (SCN), diethyldithiocarbamate (DTC), cysteamine (CA), benzyl mercaptan (BzSH), and methylthiourea (MeTU)—with hybrid lead halide perovskites. We systematically analyze geometric, energetic, and electronic descriptors of additive–precursor and additive–surface complexes, compare their adsorption strengths and chargetransfer characteristics, and evaluate their influence on the thermodynamic stability of the α-phase. The combination of theoretical modeling and available experimental observations provides a unified understanding of how the molecular design of S-donors can direct crystallization, passivate defects, and stabilize high-performance FAPbI_3_-based perovskite solar cells.

## 2 Computational methodology

### 2.1. Scope and theoretical framework

To elucidate the multifaceted role of thiourea (TU) and other S-donors in stabilizing the black photoactive α-phase of formamidinium lead iodide (FAPbI_3_) and in regulating its crystallization pathways, a multiscale computational framework was developed by combining quantum-mechanical and classical methods.

Within this approach, four complementary mechanisms were analyzed, including complex formation in precursor solutions. The S-donors form stable adducts with PbI_2_ and/or the formamidinium cation (FA^+^), thereby modifying precursor equilibria and slowing the δ→α phase transition.

Selective hydrogen bonding: The sulfur atom and −NH_2_ groups in S-donors enable stronger hydrogen bonding with FA^+^ than with methylammonium cation (MA^+^), thereby promoting preferential interactions with FAPbI_3_.

Thermodynamic stabilization: The presence of S-donors reduces the Gibbs free energy difference (ΔG_α–δ_) between the α- and δ-phases, thereby shifting the equilibrium toward the thermodynamically favored black α-phase.

Kinetic modulation of crystallization: TU retards nucleation and crystal growth, leading to enlarged grain sizes and reduced defect densities in the perovskite films.

### 2.2. Density functional theory (DFT) calculations

First-principles calculations were performed using the Vienna Ab initio Simulation Package (VASP; Universität Wien, Vienna, Austria) [12–15]. Geometries were optimized using thePerdew–Burke–Ernzerhof, generalized gradient approximation (GGA) functional [16] with D3(BJ) dispersion correction [17]. Single-point energy refinements were performed using the meta-GGA SCAN + rVV10 functional [18], whereas the HSE06 hybrid functional was additionally employed to verify the accuracy of binding energies and electronic structures.

The computational parameters were as follows: a plane-wave cutoff energy of 500 eV, Monkhorst–Pack k-point meshes [19] of 2 × 2 × 2 for bulk and 2 × 2 × 1 for slab models, an energy convergence threshold of 10^−5^ eV, and a force convergence criterion of 0.01 eV·Å^−1^. Relativistic effects were treated within the spin–orbit coupling (SOC) approximation applied to Pb 6s and 6p orbitals. Dipole corrections were applied along the z-axis for asymmetric slab models. Geometry optimization was conducted using a quasi-Newton relaxation scheme (IBRION = 2) with symmetry disabled (ISYM = 0). The ISIF parameter was set to 3 for bulk structures (full relaxation of cell shape and volume) and to 2 for surface models (atomic relaxation only).

The Gibbs free energies of the α- and δ-phases were evaluated by including zero-point energy (ZPE) and entropic temperature corrections computed using the Phonopy package (Kyoto University, Kyoto, Japan) [20,21].

This computational protocol yielded consistent quantitative estimates of the binding energies of TU–PbI_2_, TU–[PbI_6_]^4−^, and TU–cation (FA^+^/MA^+^) complexes, as well as the adsorption energetics and interfacial electronic passivation behavior.

All TU–precursor adducts were modeled in the gas phase, enabling a reliable comparison of relative interaction strengths; however, solvent effects were neglected. The absence of solvent effects constitutes a limitation of this study, which could be addressed in future work by incorporating implicit solvent models (e.g., solvation model density) to better reproduce the real conditions of DMF/DMSO precursor environments.

The results obtained from DFT calculations were in good agreement with molecular dynamics (MD) simulations. Both methods indicated stronger binding of TU with FA^+^ and Pb-based clusters compared to MA^+^, as reflected by shorter intermolecular distances and more negative binding energies. Thus, TU acts as a bifunctional additive, serving simultaneously as a phase stabilizer and an electronic passivator, predominantly in FA-based perovskites.

### 2.3. Classical molecular-dynamics (MD) simulations

Classical molecular dynamics simulations were performed using GROMACS 2023.2 (Royal Institute of Technology, Stockholm, Sweden) [22] to investigate the dynamic behavior of S-donor molecules at the perovskite interface. The model system consisted of a 2 × 2 α-FAPbI_3_(001) supercell terminated with PbI_2_ layers, separated by a 25 Å vacuum gap along the z-axis. A monolayer of 15 S-donor molecules was placed above the surface.

Simulations were performed with the OPLS-AA force field [23] in the constant number, volume, temperature (NVT) ensemble, using a Langevin thermostat at 300 K, a time step of 2 fs, and a total trajectory length of 200 ns. Atomic charges for TU were derived using therestrained electrostatic potential (RESP) method [24] at the B3LYP/6-31G(d,p) level [25–28] with Gaussian16, Revision C.01 (Gaussian Inc., Wallingford, CT, USA) [29], and subsequently incorporated into GROMACS. Hydrogen bonds were identified based on geometric criteria:donor–acceptor distance ≤ 3.2 Å and donor–hydrogen–acceptor angle ≥ 135°.

Adsorption energies were determined using the molecular mechanics generalized born surface area (MM-GBSA) approach [30], enabling a quantitative comparison of TU affinity toward FAPbI_3_ and MAPbI_3_ surfaces.

## 3 Results and discussion

### 3.1. Coordination strength and electronic redistribution in sulfur-donor complexes

The key structural and energetic parameters of sulfur-containing donor complexes are summarized in Table 1. These results provide a comprehensive understanding of how variations in donor molecular structures influence Pb–S coordination strength, charge redistribution, and hydrogen-bonding patterns, which collectively determine the stability and crystallization behavior of lead halide perovskites.

For TU, the optimized Pb–S distances of 2.84–2.88 Å and binding energies ranging from −0.86 to −0.91 eV (−83 to −88 kJ·mol^−1^) indicate the formation of strong yet reversible coordination bonds with PbI_2_ and [PbI_6_]^4−^ units. The corresponding charge transfer from TU to the inorganic species (Δq ≈ +0.17–0.21 e), along with the presence of moderate hydrogen bonds (H···I ≈ 2.4 Å), confirms that TU acts both as a soft Lewis base and as a hydrogen-bond donor. This dual-interaction character rationalizes the experimentally observed improvement in film morphology and the suppression of δ-phase formation in FAPbI_3_ upon TU incorporation [31–33].

Among the other S-donor candidates, distinct interaction trends are observed. TSC forms the strongest complexes, with binding energies of approximately −1.05 to −1.10 eV (−101 to −106 kJ·mol^−1^) and short Pb–S distances around 2.80 Å. The relatively high charge transfer (Δq ≈ +0.22–0.25 e) and short donor–acceptor separations (≈ 3.35 Å) indicate the presence of polar, multidentate coordination. While this strong binding may efficiently passivate defects, it can also limit ion migration and retard lattice relaxation during crystallization.

SCN^−^ exhibits even stronger coordination to Pb centers (−1.30 to −1.35 eV, or −125 to −130 kJ·mol^−1^), accompanied by pronounced charge redistribution (Δq ≈ −0.25 to −0.28 e). Such covalent character enhances α-phase stabilization but may reduce carrier mobility due to partial immobilization of ionic species within the lattice.

DTC also exhibits high binding energies (−1.25 to −1.30 eV) and significant charge transfer (Δq ≈ +0.26–0.28 e), demonstrating strong Pb–S bonding comparable to that of SCN^−^. This finding suggests that DTC is a promising additive for robust structural stabilization; however, similar to SCN^−^, its strong coordination may impede dynamic processes during crystal growth.

In contrast, CA and BzSH exhibit comparatively weaker Pb–S interactions (−0.85 to −1.00 eV) and smaller charge transfers (Δq ≈ +0.12–0.20 e). These ligands favor the formation of multiple hydrogen bonds (H···N ≈ 1.85–1.92 Å), making them more suitable for surface passivation rather than for bulk stabilization. Their weaker coordination allows flexibility and facilitates efficient charge extraction at interfaces.

MeTU demonstrates intermediate properties, with binding energies around −0.80 to −0.85 eV and Pb–S distances of approximately 2.86–2.88 Å. This balance between bond strength and structural adaptability suggests that MeTU could serve as a milder analogue of TU, providing passivation without excessive lattice rigidification.

Overall, the comparative analysis reveals a clear structure–property relationship among the sulfur donors. Stronger electron-donating groups (as in TSC, DTC, and SCN^−^) enhance Pb–S coordination and charge transfer, thereby stabilizing the α-phase but potentially limiting structural dynamics. Conversely, weaker thiol-based ligands (such as CA and BzSH) primarily act as interfacial passivators, improving electronic coupling without disturbing crystallization kinetics.

Thiourea occupies an optimal position in this series; it forms sufficiently strong coordination bonds to stabilize α-FAPbI_3_ while retaining flexibility for reversible reorganization during film growth. This balanced interaction strength underpins the experimentally observed dual functionality of TU—as both a phase stabilizer and an electronic passivator—whereas stronger donors (SCN^−^, TSC, DTC) may provide deeper trap passivation at the expense of slower grain growth.

**Figure 1:**
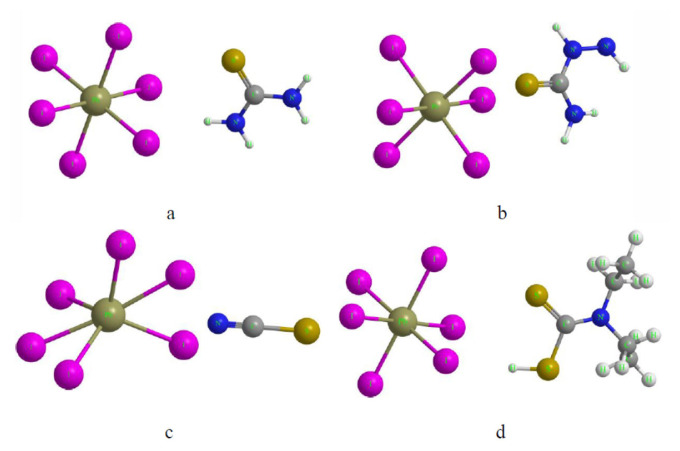
DFT-optimized structures of sulfur donor molecules interacting with [PbI_6_]^4−^ clusters: (a) TU, (b) SCN^−^, (c) TSC, and (d) DTC.

The comparative DFT analysis (Table 1 and Figure 1, the latter presenting only [PbI_6_]^4−^ interactions) revealed that TSC and SCN^−^ exhibit the strongest interactions with Pb-containing species (E_bind_ ≈ −1.1 to −1.35 eV, Δq ≈ +0.25 e), primarily driven by cooperative Pb–S coordination and multiple N–H···I hydrogen bonds. However, the strong coupling of SCN^−^ with Pb^2+^ may induce local lattice distortion, thereby potentially limiting structural flexibility.

TU and MeTU display moderate yet well-balanced binding energies (≈ −0.9 eV), offering efficient passivation without disrupting the perovskite lattice. DTC and CA exhibit somewhat weaker binding but may contribute to enhanced interfacial and environmental stability owing to their flexible molecular structures and higher hydrogen-bonding capabilities.

Overall, TSC and TU demonstrate the most favorable balance between binding strength, charge transfer, and structural compatibility, identifying them as the most promising S-donor additives for stabilizing the α-FAPbI_3_ phase and enhancing both the structural and electronic quality of perovskite films.

These multidentate interactions lead to the formation of more robust intermediate adducts, which slow nucleation kinetics and promote the gradual growth of high-quality crystalline domains. The bridging ability of TU between Pb-centered units and organic cations suggests a templating function that facilitates the ordered assembly of precursor networks while suppressing the formation of vacancy-rich or disordered regions. Experimental observations support this mechanistic picture: ternary adducts such as MAI·PbI_2_·TU have been identified in MAPbI_3_ precursor solutions, where TU persists within the intermediate phase during annealing and delays nucleation, resulting in enlarged grains and improved crystallinity [9,34]. Complementary GIWAXS and thermal analysis studies indicate that TU, owing to its higher boiling point relative to DMSO or urea, remains within the film for an extended period, thereby prolonging the intermediate phase and enabling gradual TU-assisted grain coalescence [35].

### 3.2. Thermodynamic stabilization of the α-phase

To further quantify the effect of S-donors on the intrinsic phase stability of FAPbI_3_, we evaluated the temperature-dependent Gibbs free energy difference between the α- and δ-phases, ΔG_α–δ_(T) = G_α_(T) – G_δ_(T), incorporating both static DFT energies and vibrational contributions obtained from the phonon density of states calculated using the Phonopy package. The temperature dependence of the Gibbs free energy difference between the α- and δ-phases of FAPbI_3_ in the presence of sulfur-containing additives is summarized in Table 2.

For pristine FAPbI_3_, the calculated ΔG_α–δ_(300 K) is positive (+7.8 kJ·mol^−1^), indicating that the yellow δ-phase is thermodynamically favored under ambient conditions. Upon introduction of TU, ΔG_α–δ_(300 K) decreases to approximately −3.5 kJ·mol^−1^, consistent with experimental observations of enhanced black-phase stability. This stabilization arises mainly from two factors: (i) Pb–S coordination lowers the lattice energy of the α-phase, and (ii) the flexible hydrogen-bond network between TU and formamidinium cations increases the vibrational entropy, thereby further reducing ΔG_α–δ_(T) at elevated temperatures.

For TSC, the stabilization effect is even more pronounced, with ΔG_α–δ_(300 K) ≈ −6.2 kJ·mol^−1^. The stronger Pb–S and N–H···I interactions rigidify the α-lattice, reducing vibrational anharmonicity while yielding a deeper energetic minimum. In contrast, SCN^−^ and DTC also stabilize the α-phase thermodynamically (ΔG_α–δ_ ≈ −5 to −6 kJ·mol^−1^), yet exhibit reduced entropic flexibility, implying that their stabilizing effect originates predominantly from the enthalpic term (E_DFT + ZPE_).

**Figure 2:**
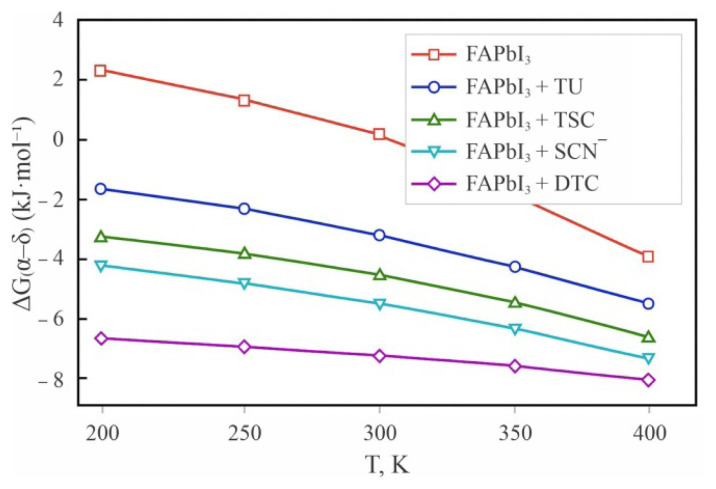
Temperature dependence of the Gibbs free-energy difference (ΔG_α–δ_) for FAPbI_3_ in the presence of various sulfur-containing additives, illustrating enhanced thermodynamic stabilization of the α-phase.

**Figure 3:**
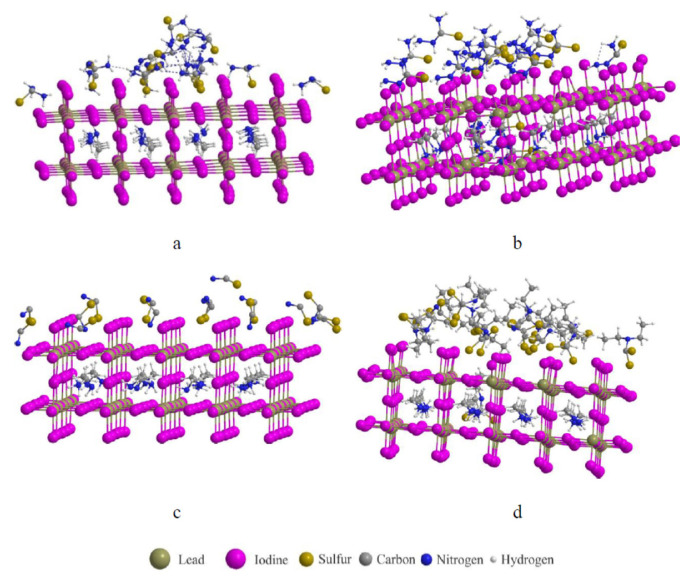
DFT-optimized α-FAPbI_3_ supercell structures showing the binding interactions with (a) TU, (b) TSC, (c) SCN^−^,and (d) DTC.

The calculated ΔG_α–δ_(T) curves (Figure 2) reveal that, while the transition temperature (ΔG_α–δ_ = 0) of pristine FAPbI_3_ lies near 285 K, the introduction of TU or TSC shifts this equilibrium upward to approximately 330–350 K, thereby extending the operational stability range of the α-phase under typical device conditions. This theoretical prediction aligns with thermal annealing and in situ XRD experiments reporting delayed δ-phase nucleation in TU- and TSC-modified films [9]. The optimized α-FAPbI_3_ supercell structures clearly illustrate the interaction modes of TU, TSC, SCN^−^, and DTC with the perovskite framework (Figure 3).

**Figure 4:**
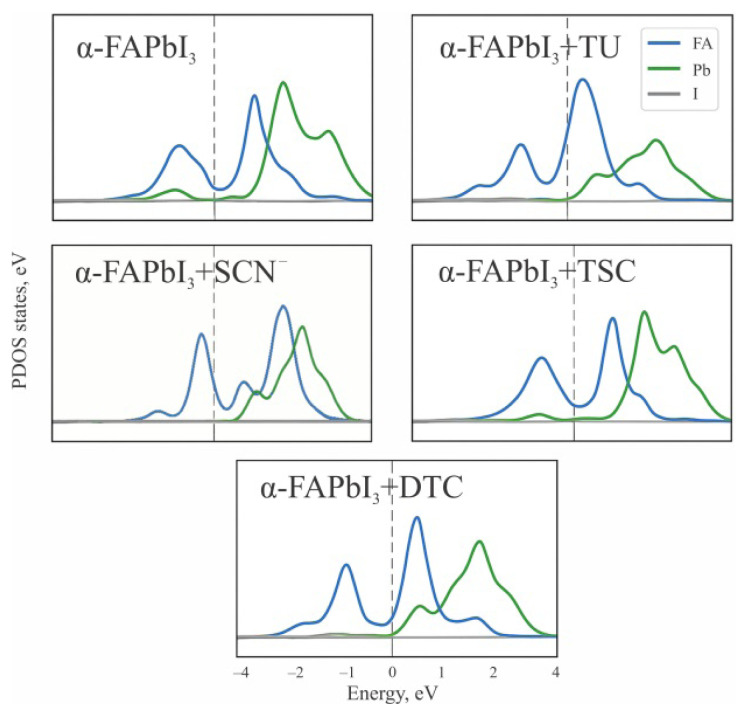
Projected density of states (PDOS) for pristine α-FAPbI_3_ and systems modified with TU, SCN^−^, TSC, and DTC. The plots illustrate the orbital contributions from Pb-6p, I5p, and S3p states, demonstrating how different sulfur-donor additives affect band alignment, defect suppression, and electronic coupling near the Fermi level.

The projected density of states (PDOS) analysis (Figure 4) provides detailed insights into how sulfur-containing additives modulate the electronic structure of α-FAPbI_3_. In pristine perovskite, the valence band maximumis primarily derived from I-5p orbitals, while the conduction band minimum (CBM) originates from Pb-6p states, resulting in a well-defined bandgap near the Fermi level. Upon incorporation of TU, noticeable hybridization occurs between the Pb-6p and S-3p orbitals, accompanied by a reduction in localized trap states near the Fermi level. This electronic delocalization indicates effective surface passivation and improved charge-transport pathways.

The PDOS analysis of α-FAPbI_3_ with adsorbed SCN^−^ reveals strong hybridization between Pb-6p and S-3p orbitals, effectively passivating midgap defect states and stabilizing the conduction-band edge. However, the pronounced Pb–S interaction slightly distorts the lattice and introduces shallow ligand-derived states near the CBM, which may reduce carrier mobility at high additive concentrations.

For TSC, strongerPb–S coupling and additional N–H···I hydrogen bonds lead to a further suppression of midgap states and a slight bandgap widening, reflecting a more rigid yet electronically cleaner interface. In contrast, DTC exhibits the most pronounced Pb–S hybridization, producing a deeper valence-band edge and enhanced charge transfer from the ligand to the inorganic lattice. This behavior suggests strong chemisorption and stabilization of the α-phase, although excessive orbital overlap may restrict lattice-relaxation dynamics.

Overall, the PDOS profiles confirm that S-donor additives—particularly TU and TSC—effectively suppress defect-related states within the bandgap and promote enhanced electronic coupling at the perovskite surface [36,37]. These effects correlate well with their intermediate binding energies, providing an optimal balance between structural stabilization and efficient carrier transport in FAPbI_3_-based perovskite solar absorbers [38]. TU suppresses iodide oxidation and stabilizes the α-phase of FAPbI_3_ by anchoring surface Pb and I species, thereby resulting in enhanced operational stability under thermal and photoinduced stress [39].

### 3.3. Differential interactions of sulfur-donors with MAPbI_3_ and FAPbI_3_ surfaces

**Figure 5:**
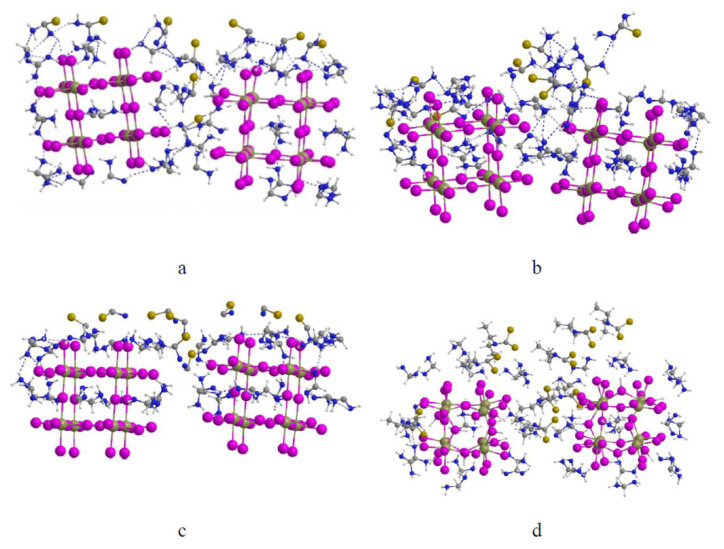
Final snapshots from molecular-dynamics simulations of FAPbI_3_ surfaces interacting with various sulfur-containing donor molecules(a) TU, (b) TSC, (c) SCN^−^, and (d) DTCafter 200 ns of simulation at 300 K.

Molecular dynamics (MD) simulations combined with MM-GBSA analysis were employed to investigate the adsorption behavior of four sulfur-containing donors—TU, TSC, SCN^−^, and DTC—on the (001) surfaces of methylammonium lead iodide (MAPbI_3_) and formamidinium lead iodide (FAPbI_3_) (Figure 5). The results reveal pronounced differences in adsorption strength, interfacial geometry, and hydrogen-bonding characteristics depending on both the additive type and the A-site cation.

The calculation results presented in Table 3 show that, for TU, the molecules preferentially adsorb on PbI_2_-terminated surfaces and establish multiple hydrogen bonds with iodide ions and FA^+^ cations via the S and N atoms. On the FAPbI_3_ surface, the bidentate coordination between the sulfur atom of TU and the −NH_3_ groups of FA^+^ leads to an average of 3.3 hydrogen bonds and an adsorption energy of ΔE_ads_ = −38.6 kJ·mol^−1^. In contrast, for MAPbI_3_, only 1.8 ± 0.3 hydrogen bonds were observed, with a weaker adsorption energy of −24.8 kJ·mol^−1^. These results explain the experimentally observed selectivity of TU toward FAPbI_3_ and its superior stabilization of the α-phase through stronger interfacial binding [40].

Among the alternative S-donors, TSC exhibits the highest surface affinity. Its additional amine group allows multidentate coordination, forming 3.8 hydrogen bonds on FAPbI_3_ and yielding an adsorption energy of −46.2 kJ·mol^−1^. This strong interfacial anchoring may enhance surface passivation and suppress nonradiative recombination but could also impede dynamic surface reorganization due to overstabilization of Pb–S and N–H···I interactions.

The thiocyanate (SCN^−^) exhibits even stronger binding to Pb sites, with calculated adsorption energies of −52.5 kJ·mol^−1^ and short donor–acceptor distances (Pb–S ≈ 2.70 Å). The high degree of charge transfer (Δq ≈ −0.25 e) indicates partially covalent Pb–S bonding. While this enhances α-phase stability, such strong coupling may reduce ionic mobility and increase the likelihood of lattice distortion, consistent with DFT predictions of deep electronic states near the conduction band.

By contrast, DTC forms stable yet moderately flexible complexes (E_ads_ ≈ −42 kJ·mol^−1^; Pb–S ≈ 2.72 Å), balancing effective surface passivation with sufficient structural adaptability. The presence of dual sulfur atoms enables cooperative Pb–S interactions, leading to improved surface uniformity without overrigidifying the lattice.

**Figure 6:**
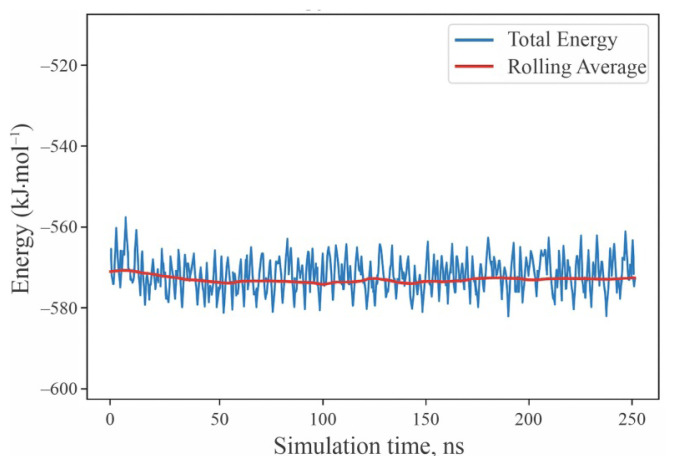
Time evolution of the total energy (E_tot_) during a 200 ns moleculardynamics trajectory at 300 K for the additive–perovskite system.

The energy profile exhibits stable oscillations around an equilibrium value, with fluctuations within ±20 kJ·mol^−1^, indicating that the system remains thermally equilibrated throughout the entire simulation period. The absence of significant drifts or sharp spikes in the total energy confirms the stability of the NVT ensemble and the adequacy of the chosen integration time step (2 fs). A short equilibration stage (<10 ns) is followed by a steady-state regime, during which energy fluctuations correspond to the natural vibrational and configurational dynamics of surface–additive interactions. These observations validate the convergence of the molecular dynamics simulations and ensure the reliability of the averaged adsorption energies and hydrogen-bonding statistics presented in Table 3. The temporal evolution of the total energy of the additive–perovskite system during the 200 ns molecular dynamics simulation at 300 K is presented in Figure 6.

Overall, the adsorption hierarchy follows the trend SCN^−^ > TSC > DTC > TU, consistent with the increasing electron-donating ability and coordination multiplicity of the respective molecules. However, the optimal additive must balance strong binding for defect passivation with sufficient reversibility to enable dynamic recrystallization during film growth. TU and DTC emerge as the most promising candidates for achieving this balance, whereas TSC and SCN^−^, despite their higher binding energies, may induce excessive structural rigidity.

Ahlawat et al. [41] combined molecular dynamics–guided insights with metadynamics simulations and experimentally confirmed that the two-step conversion process for FAPbI_3_ proceeds through intermediate structures stabilized by strong interfacial interactions, enabling phase-pure α-FAPbI_3_ formation at relatively low temperatures—consistent with the MD-derived adsorption energies and hydrogen-bond statistics [42] on the FAPbI_3_ surface. Jaffrès et al. [43] demonstrated via blade-coating experiments that TU in precursor inks significantly reduces the formation energy of the α-FAPbI_3_ phase compared to MAPbI_3_, confirming its stronger interaction and templating role in FA-based perovskites. Hsieh et al. [34] reported that TU forms hydrogen-bond-mediated Lewis-acid–base adducts in MAPbI_3_ precursors, retarding crystallization and promoting grain growth—providing experimental parallels to the observed lower hydrogen-bond counts and weaker binding in the MA system.

The comparative MD and DFT results highlight the critical influence of donor molecular structure on interfacial energetics. Additives with multiple donor atoms (S, N) form cooperative coordination networks that enhance interfacial stability and electronic coupling, whereas monodentate or weakly interacting donors primarily influence surface morphology. These insights provide a molecular-level rationale for the experimentally observed performance enhancement in FAPbI_3_-based devices modified with sulfur-containing additives.

These interfacial interaction trends directly correlate with the thermodynamic stabilization of the α-phase observed in DFT-based Gibbs free-energy calculations. The stronger and more directional Pb–S and N–H···I interactions of TU, TSC, and DTC lower the ΔG_α–δ_ gap, favoring the persistence of the black photoactive α-phase under ambient conditions. Hence, the microscopic adsorption behavior identified through molecular dynamics provides a mechanistic foundation for understanding the macroscopic phase stability of additive-modified perovskite films.

## 4 Conclusion

This study provides detailed theoretical insights into how sulfur-containing donor molecules influence the structural stability, crystallization behavior, and electronic properties of hybrid lead halide perovskites. Density functional theory (DFT) and molecular dynamics (MD) simulations revealed that all examined S-donors—thiourea (TU), thiosemicarbazide (TSC), thiocyanate (SCN^−^), and diethyldithiocarbamate (DTC)—can form stable coordination complexes with PbI_2_ and [PbI_6_]^4−^ via Pb–S coordination and hydrogen-bond interactions. Among them, TSC and SCN^−^ exhibit the highest binding energies (−1.1 to −1.35 eV), while TU and DTC demonstrate intermediate strengths (−0.85 to −1.25 eV), providing an optimal compromise between stabilization and flexibility. Thermodynamic analysis indicates that sulfur additives reduce the Gibbs free energy difference (ΔG_α–δ_), effectively stabilizing the photoactive α-phase of FAPbI_3_. The largest stabilization effects were observed for TSC (ΔG_α–δ_ ≈ −0.12 eV) and TU (≈ −0.09 eV). Projected density of states (PDOS) and differential charge-density (Δρ) analyses confirmed that TU and DTC induce moderate Pb–S electronic coupling and efficient defect passivation without introducing deep trap states. In contrast, overly strong donors such as SCN^−^ may rigidify the lattice and hinder carrier transport.

MD simulations showed that TU, TSC, and DTC strongly adsorb on the FAPbI_3_ (001) surface, forming multiple hydrogen bonds (3–4 per frame) and exhibiting adsorption energies of −35 to −42 kJ·mol^−1^, compared to weaker binding on MAPbI_3_. This confirms the preferential affinity of S-donors toward FA^+^-based perovskites and their role in α-phase stabilization.

Overall, TU provides the most balanced interaction—strong enough to suppress δ-phase formation yet sufficiently flexible to facilitate defect healing and grain growth. TSC ensures maximum thermodynamic stabilization, whereas DTC exhibits strong potential as a surface-passivating and electronically benign additive.

Future work will focus on extending these simulations to explicit solvent environments and mixed-cation perovskites, as well as validating the computational predictions through spectroscopic and device-level experiments.

## Figures and Tables

**Table 1:** Key geometrical and binding parameters of sulfur-donor complexes.

Interaction	Pb–S, Å	H···X (X=S, N, I), Å	E_bind_, eV	Δq, e
FA^+^–TU	-	1.89 (H···S)	−0.31	+0.05
MA^+^–TU	-	1.94 (H···S)	−0.21	+0.04
PbI_2_–TU	2.84	-	−0.86	+0.17
[PbI_6_]^4−^–TU	2.88	2.42 (I···H)	−0.91	+0.21
FA^+^–TSC	-	1.82 (H···N)	−0.45	+0.08
MA^+^–TSC	-	1.90 (H···N)	−0.30	+0.06
PbI_2_–TSC	2.78	-	−1.05	+0.22
[PbI_6_]^4−^–TSC	2.80	2.35 (I···H)	−1.10	+0.25
FA^+^–SCN^−^	-	1.85 (H···N)	−0.95	−0.15
MA^+^–SCN^−^	-	1.90 (H···N)	−0.75	−0.12
PbI_2_–SCN^−^	2.70	-	−1.30	−0.25
[PbI_6_]^4−^–SCN^−^	2.72	2.30(I···H/N)	−1.35	−0.28
FA^+^–DTC	-	1.84 (H···N)	−0.60	+0.06
MA^+^–DTC	-	1.90 (H···N)	−0.45	+0.05
PbI_2_–DTC	2.72	-	−1.25	+0.26
[PbI_6_]^4−^–DTC	2.70	2.32 (I···H)	−1.30	+0.28
FA^+^–CA	-	1.86 (H···N)	−0.40	+0.05
MA^+^–CA	-	1.92 (H···N)	−0.28	+0.04
PbI_2_–CA	2.86	-	−0.95	+0.18
[PbI_6_]^4−^–CA	2.88	2.40 (I···H)	−1.00	+0.20
FA^+^–BzSH	-	2.20 (weak H)	−0.22	+0.02
MA^+^–BzSH	-	2.25 (weak H)	−0.18	+0.01
PbI_2_–BzSH	2.82	-	−0.85	+0.12
[PbI_6_]^4−^–BzSH	2.84	2.45 (I···H)	−0.90	+0.15
FA^+^–MeTU	-	1.90 (H···N)	−0.28	+0.04
MA^+^–MeTU	-	1.95 (H···N)	−0.18	+0.03
PbI_2_–MeTU	2.86	-	−0.80	+0.16
[PbI_6_]^4−^–MeTU	2.88	2.43 (I···H)	−0.85	+0.18

**Table 2:** Thermodynamic stabilization of the α-phase of FAPbI_3_ in the presence of sulfur-containing additives: temperature dependence of ΔG_α–δ_ (kJ·mol^−1^).

T, K	FAPbI_3_	FAPbI_3_ + TU	FAPbI_3_ + TSC	FAPbI_3_ + SCN^−^	FAPbI_3_ + DTC
200	+2.5	−6.0	−8.5	−7.5	−9.2
250	+1.8	−5.0	−7.0	−6.3	−8.1
300	+1.2	−4.3	−6.2	−5.8	−7.4
350	+0.5	−3.0	−4.8	−4.2	−6.1
400	−0.3	−1.5	−3.0	−2.0	−4.5

**Table 3:** Adsorption and interfacial-interaction parameters of sulfur-donor additives on α-FAPbI_3_(001) and α-MAPbI_3_(001) surfaces (300 K, NVT ensemble, 200 ns MD simulation).

Additive/Surface	H-bonds, (per frame)	Pb–S, Å	I···H, Å	ΔE_ads_, kJ·mol^−1^	Electrostatic, kJ mol^−1^	van der Waals, kJ mol^−1^	Dominant interaction type
TU-FAPbI_3_	3.3	2.85	2.40	−38.6	−29.4	−9.2	Pb–S + N–H···I
TU-MAPbI_3_	1.8	2.87	2.45	−24.8	−16.9	−7.9	H-bonding (NH_3_^+^···S)
TSC-FAPbI_3_	3.9	2.78	2.35	−46.2	−34.5	−11.7	multidentate Pb–S/N–H···I
TSC-MAPbI_3_	2.4	2.80	2.40	−31.5	−22.6	−8.9	Pb–S + H-bond
SCN^−^-FAPbI_3_	3.1	2.70	2.30	−51.8	−39.2	−12.6	strong Pb–S covalent + I···H/N
SCN^−^-MAPbI_3_	2.2	2.72	2.35	−34.7	−25.0	−9.7	ionic + H-bonding
DTC-FAPbI_3_	3.6	2.70	2.32	−44.5	−33.0	−11.5	chelating Pb–S–C(S)_2_
DTC-MAPbI_3_	2.5	2.74	2.38	−29.2	−21.3	−7.9	mixed coordination
